# Supplementary material for: Association between chronic physical conditions and depressive symptoms among hospital workers in a national medical institution designated for COVID-19 in Japan
Source: PLoS One. 2022 Apr 7;17(4):e0266260. doi: 10.1371/journal.pone.0266260 (PMC8989319; doi:10.1371/journal.pone.0266260)
Supplement: S1 Table — (DOCX) [file pone.0266260.s001.docx]

**S1 Table. Information on chronic physical conditions (n=486)^*^.**

| **Chronic physical conditions** | **n** |
| --- | --- |
| Hypertension | 166 |
| Chronic obstructive pulmonary disease (COPD) or bronchial asthma | 95 |
| Diabetes | 55 |
| Cardiovascular diseases | 38 |
| Cancers | 29 |
| Cerebrovascular diseases | 16 |
| Other chronic physical conditions  (e.g., dyslipidemia, thyroid disorders, anemia, human immunodeficiency (HIV) infection, neurological disorders, and autoimmune diseases) | 246 |

^*^110 participants had more than one conditions.
