# Supplementary material for: Association between chronic physical conditions and depressive symptoms among hospital workers in a national medical institution designated for COVID-19 in Japan
Source: PLoS One. 2022 Apr 7;17(4):e0266260. doi: 10.1371/journal.pone.0266260 (PMC8989319; doi:10.1371/journal.pone.0266260)
Supplement: Attachment — Submitted filename: Response Letter_comobdep_Fukunaga_final.docx [file pone.0266260.s002.docx]

March 8th, 2022

Dr. Emily Chenette

Editor-in-Chief, PLOS ONE

Dr. Stephan Doering

Academic Editor, PLOS ONE

Manuscript No.:	PONE-D-21-28081

Title:	Association between chronic physical conditions and depressive symptoms among hospital workers in a national medical institution designated for COVID-19 in Japan

Dear Dr. Emily Chenette and Dr. Stephan Doering,

We would like to thank you for offering us an opportunity to revise the above-mentioned manuscript. Following the comments and suggestions from the editor and reviewers, we have revised the manuscript. We are now submitting the revised version of the manuscript. All the changes in the manuscript are marked in red, and details of our responses to the reviewers’ comments are described in detail as following.

Thank you for your consideration of our manuscript for publication.

Sincerely yours,

Ami Fukunaga

Department of Epidemiology and Prevention

National Center for Global Health and Medicine, Tokyo, Japan

1-21-1 Toyama, Shinjuku-ku, Tokyo, 162-8655, Japan

Tel: +81-3-3202-7181   Ext. 2859

Fax: +81-3-3202-7364

E-mail: afukunaga@hosp.ncgm.go.jp

**Authors’ Responses to the Reviewers**

**Reviewer #1:**

This study of chronic physical conditions and depression among over 2000 healthcare workers at a COVID-19 treatment center in Japan provides important insight into the drivers of mental health issues during COVID-19 for this population. The methods are strong and the manuscript is well written.

We greatly appreciate all the constructive comments and suggestions. We have revised the manuscript in accordance with your comments and suggestions.

METHODS

Q1-1. Ln 42. It’s not clear why participants who reported depression, hay fever, or low back pain were excluded, is it just because these were not the chronic physical conditions of interest? If so, I would frame in terms of inclusion rather than exclusion criteria.

A1-1. Thank you for raising this important issue. As the reviewer pointed out, we picked out chronic physical conditions that were known to be risk factors of severe COVID-19 symptoms or mortality to define the exposure, and the above-mentioned diseases were not the focus of this paper. In the original submission, we excluded those with the existing conditions because such conditions might have occurred and resulted in depression before the COVID-19 pandemic.

This comment, however, has helped us realize that we should not have excluded those with hay fever or low back pain as these conditions were not strong determinants of depression. As regards those with depression, we decided to keep the original exclusion criteria as existing depression at the early phase of the pandemic might not have been caused by the fear that physical conditions put them at higher risk of severe COVID-19 symptoms.

As a response to this comment, we have clarified the definition of chronic physical conditions and the study participant selection process. With this change, the total number of participants changed from 2337 to 2400. We confirmed that results of all analyses did not change from the previous version. The following changes were made to the manuscript:

Line 46–48: In addition, we excluded those who reported having depression (n=5) as it is possible that they experienced the outcome before the onset of COVID-19 pandemic.

Line 54–64: In this study, we defined the presence of chronic physical conditions using information on chronic physical conditions that have been suggested to increase risk of severe COVID-19 outcome [5, 6]. We asked participants whether they have any of the following chronic physical condition(s): diabetes, hypertension, chronic obstructive pulmonary disease (COPD) or bronchial asthma, cardiovascular disease, cerebrovascular disease, cancer, or other chronic physical conditions. The response options for each condition included “no,” “yes (under medication),” and “yes (untreated)” (plus “yes [diet therapy]” for diabetes). For other chronic physical conditions, participants were asked to specify the conditions. From the list of other conditions, we picked out the following chronic physical conditions that meet our definition of exposure: dyslipidemia [9], thyroid disorders [10], anemia [11], human immunodeficiency virus (HIV) infection [6], neurological disorders [12], and autoimmune diseases [13].

Q1-2. Ln 99-101. Please provide more context for the inclusion of these questions about being bad-mouthed or discriminated against. Is this because they work in a hospital designated for COVID-19 treatment? t’s not clear how this is COVID-related or how it’s relevant to the research question. If there is a culturally specific rationale, it would be helpful to explain this.

A1-2. We appreciate this valuable suggestion and we are sorry for our poor description. The questions on being bad-mouthed and discrimination were both related with the COVID-19. As our participants worked in a national medical institution designated for COVID-19 treatment, they were deemed at a higher risk of COVID-19-related discrimination and thus, psychological stress as exemplified in a recent meta-analysis by Schubert et al. that has linked such experience to a worsening of mental health. Thus, these questions were considered when we account for the association between chronic physical condition and depressive symptoms. In fact, those with chronic physical conditions in this study were more likely to experience COVID-19-related discrimination (13.2%) than those without any condition (8.7%) and we would like to adjust this difference in the regression.

As a response to this comment, we explained the rationale for the inclusion of these questions as shown below.

Line 108–112: Regarding participants’ experiences in relation to COVID-19, we asked them if they agreed with the following two statements: “You and your family have been bad-mouthed” (yes or no) and “I felt that I was discriminated against in some way” (yes or no). If participants answered “yes” to either question, they were considered as having experiences of being bad-mouthed or discriminated against in relation to COVID-19.

Line 112–114: We incorporated the discrimination experience in the present study because such experiences have been suggested to worsen mental health [16].

Reference #16: Schubert M, Ludwig J, Freiberg A, Hahne TM, Romero Starke K, Girbig M, Faller G, Apfelbacher C, von dem Knesebeck O, Seidler A. Stigmatization from Work-Related COVID-19 Exposure: A Systematic Review with Meta-Analysis. Int J Environ Res Public Health 2021, 18(12):6183.

DISCUSSION

Q1-3. It would be good to place these findings in context with the physical and mental health of the Japanese population more generally.

A1-3. We greatly appreciate this valuable suggestion. Following this suggestion, we added a paragraph in which we discussed our findings in context with the physical and mental health of the Japanese population as shown below.

Line 207–221: The present as well as previous studies conducted among the general population [17, 18] provide robust evidence on the association between chronic physical conditions and depression/depressive symptoms during the COVID-19 pandemic. Given a growing number of ageing population and people with chronic physical conditions in Japan [27] as well as other countries [28], the association should be more extensively studied. It is possible that people with chronic physical conditions experience depressive symptoms as they might have fear of being infected and developing severe COVID-19 outcome or they might isolate themselves and make adverse changes in lifestyle behaviors (e.g., decreased physical activity, alcohol drinking) as a consequence of perceiving the risk and stress associated with having chronic physical conditions. Such psychological stress might further exacerbate their physical conditions and affect mental health. Thus, this finding underscores the urgency that we should be aware of their conditions and pay further attention for both physical and psychological care management and treatment for people with chronic physical conditions.

Q1-4. Also, how is mental health conceptualized in Japan more broadly? Is there a lot of stigma that could result in underreporting, particularly in the healthcare worker population?

A1-4. We would like to thank the reviewer for raising these important issues. Japanese people are aware that the country has faced tremendous disease burden associated with mental health. In particular, depression and suicide are considered major social issues among working-age populations. While people used to regard mental illness as a sign of weakness, people now have a better understanding of what causes depression.

While mental-health-related stigma could have still existed in Japan, depressive symptoms in the present study were not likely to be subject to underreporting for the following reasons. The present study was conducted as a part of the questionnaire survey of an epidemiological study measuring SARS-CoV-2 antibody. The study was conducted by an independent investigation team. Participants were informed that their supervisors were not involved in the data collection and that t questionnaire results, including those for depressive symptoms, were anonymized and were not reported to their supervisors.

**Reviewer #2:**

Thanks for the opportunity to review the manuscript "Association between chronic physical conditions and depressive symptoms among hospital workers in a national medical institution designated for COVID-19 in Japan". The work deals with an interesting topic. However, I think some adjustments are needed before publication. I hope you find my comments useful.

We greatly appreciate all the constructive comments and suggestions. Following the comments and suggestions, we have revised the manuscript as following.

Q2-1. I would rewrite the abstract to provide a more concise and detailed idea of your work. Specify how the influence of the risk of SARS-CoV-2 infection has been considered. Try replacing "workers with the condition" with something clearer. Also, I would write from October to December 2020 instead of “or”.

A2-1. We greatly appreciate your suggestion. As per this suggestion, we modified the abstract to make it read better.

**Abstract**

**Objective**

This study aimed to investigate the cross-sectional association between the presence of chronic physical conditions and depressive symptoms among hospital workers at a national medical institution designated for COVID-19 treatment in Tokyo, Japan. We also accounted for the combined association of chronic physical conditions and SARS-CoV-2 infection risk at work in relation to depressive symptoms, given that occupational infection risk might put additional psychological burden among those with chronic physical conditions with risk of severe COVID-19 outcome.

**Methods**

The study sample consisted of 2440 staff members who participated in a health survey conducted at the national medical institution during period between October and December 2020. Participants who reported at least one chronic physical condition that were deemed risk factors of severe COVID-19 outcome were regarded as having chronic physical conditions. Depressive symptoms were assessed using the patient health questionnaire-9 (PHQ-9). We performed logistic regression analysis to assess the association between chronic physical conditions and depressive symptoms.

**Results**

Our results showed that the presence of chronic physical condition(s) was significantly associated with depressive symptoms (odds ratio (OR)=1.49, 95% confidence interval (CI)=1.10–2.02). In addition, the prevalence of depressive symptoms was significantly higher among healthcare workers with chronic physical condition(s) who were at a higher occupational infection risk (OR=1.81, 95% CI=1.04–3.16).

**Conclusion**

Our findings suggest the importance of providing more assistance to those with chronic physical conditions regarding the prevention and control of mental health issues, particularly among frontline healthcare workers engaging in COVID-19-related work.

Q2-2. The introduction section should be recasted. The authors need to provide a theoretical background on the relationship between chronic physical conditions and depressive symptoms, which theories and theoretical frameworks can justify the association and explain the underlying mechanisms (e.g., loss of autonomy, social isolation, redefinition of one's identity etc.), previous studies on the topic trying to focus on some of the specific conditions of the sample. Then, try to contextualize everything with reference to the COVID-19 emergency, I think this aspect should be emphasized also to provide a novelty. If you hypothesize that the risk of SARS-CoV-2 infection has an influence on the relationship between physical and depressive symptoms, try to justify why.

A2-2. We would like to thank the reviewer for valuable suggestion. Following your suggestions, we reconstructed the introduction section starting with general and theoretical background on the association between chronic physical conditions and depressive symptoms. In addition, we clarified our hypothesis that the occupational risk of SARS-CoV-2 infection has an influence on the relationship between chronic physical conditions and depressive symptoms. More specifically, we mentioned that health care workers with chronic physical conditions who are at higher occupational infection risk (e.g., working for infected patients) may have higher psychological distress as they are more susceptible to SARS-CoV-2 infection and adverse health outcomes as result of the infection. The modified version of the introduction is shown below.

Line 1–29:

**Introduction**

Chronic physical conditions have been studied extensively in relation to mental health [1-3]. For example, the WHO World Health Survey reported a higher prevalence of depression in people with at least one chronic physical condition (9.3–23%) compared to those without any condition (3.2%) [3]. While chronic physical conditions and depression often coexist and their association can be bidirectional [4], one line of research has suggested that chronic physical conditions cause depression/depressive symptoms [1, 2] via physical symptoms (e.g., pain and functional impairment), decreased quality of life, fear of disease progression, burden of disease self-management, and medical costs [4].

Investigation on the link between chronic physical conditions and mental health is particularly important among healthcare workers during the current pandemic of coronavirus disease 2019 (COVID-19) given that chronic physical conditions, such as hypertension and diabetes, have been suggested as risk factors of severe COVID-19 symptoms or mortality [5, 6] and that they are at a substantial risk of the severe acute respiratory syndrome coronavirus 2 (SARS-CoV-2) infection and may perceive a higher vulnerability. More specifically, frontline health care workers with chronic physical conditions who are at higher occupational infection risk (e.g., having direct contact with infected patients) may be at a higher risk of depression as they are more susceptible to SARS-CoV-2 infection and adverse health outcomes as result of the infection [5, 6]. However, we are not aware of any studies investigating the association between chronic physical conditions and mental health issues among healthcare workers. In addition, no study has investigated this subject with consideration of occupational infection risk.

The present study was designed to investigate the cross-sectional association between chronic physical conditions and depressive symptoms among hospital workers at a national medical institution designated for COVID-19 treatment in Japan. We also accounted for the combined association of the presence of chronic physical conditions and occupational infection risk in relation to depressive symptoms. We hypothesized that having chronic physical conditions might be associated with depressive symptoms, and that the magnitude of the association might be larger for individuals with chronic physical conditions who are at a higher occupational risk of SARS-CoV-2 infection.

Q2-3. Line 16. You should put the reference about the scarcity of the studies.

A2-3. We greatly appreciate this comment. We are not aware of any studies investigating the association between chronic physical conditions and mental health issues specifically among healthcare workers. Thus, we modified the descriptions in the line to explain about the lack of study.

Line 19–21: However, we are not aware of any study investigating the association between chronic physical conditions and mental health issues among healthcare workers.

Q2-4. In the methods sections you write chronic diseases, I think this definition (instead of physical symptoms) should be used even in the abstract/introduction and throughout the paper.

A2-4. We are sorry about this inconsistency in the exposure description within the manuscript. We made sure that we now use the term “chronic physical condition” when we refer to our exposure (i.e., conditions that might be risk factors for severe COVID-19 symptoms or mortality).

We avoided using the term “chronic diseases” because “chronic diseases” can be defined more broadly and may include mental disorders (i.e., outcome of the study) and “chronic diseases” may not include obesity, which was included as one of the conditions for an additional analysis. For this reason, we decided to use the term “chronic physical condition” in the present study. We revised the description in the methods sections to make the description consistent throughout the paper.

Line 55–61: We asked participants whether they have any of the following chronic physical conditions: diabetes, hypertension, chronic obstructive pulmonary disease (COPD) or bronchial asthma, cardiovascular disease, cerebrovascular disease, cancer, or other chronic physical conditions. The response options for each condition included “no,” “yes (under medication),” and “yes (untreated)” (plus “yes [diet therapy]” for diabetes). For other chronic physical conditions, the participants were asked to specify the conditions.

Q2-5. Try to explain more clearly how you classified the groups based on the degree of possible exposure to SARS-CoV-2, I understood the process but I think the authors could rewrite it to explain it better.

A2-5. We are sorry for our poor description and we would like to thank the reviewer for pointing out this clarification point. Following your suggestion, we modified sentences about the classification of the groups based on the degree of possible exposure to SARS-CoV-2 as following.

Line 101–107: In relation to engagement in COVID-19-related work, we asked the following two questions: “Have you ever engaged in COVID-19-related work?” (yes or no) and “Did you engage in any work in which you were heavily exposed to SARS- CoV-2?” (yes or no). Using these two questions, we defined the degree of possible exposure to SARS-CoV-2 at work, which was categorized into three groups: low (those who did not engage in COVID-19-related work), moderate (those engaged in COVID-19-related work without high exposure to the virus), and high (those engaged in COVID-19-related work with high exposure to the virus).

Q2-6. I suggest modifying the discussion section to better explain the findings by describing how they fit into the context of the previous literature and directions for future research. Consider adding some clinical and practical implications.

A2-6. We greatly appreciate this insightful suggestion. Following your suggestion, we added a paragraph in the discussion section to explain the findings of present and previous studies on the association between chronic physicals conditions and depression/depressive symptoms by providing some clinical and practical implications.

Line 207–221: The present as well as previous studies conducted among the general population [17, 18] provide robust evidence on the association between chronic physical conditions and depression/depressive symptoms during the COVID-19 pandemic. Given that there has been a growing number of ageing population and thus, people with chronic physical conditions in Japan [27] as well as other countries [28], the association should be more extensively studied. It is possible that people with chronic physical conditions experience depressive symptoms as they might have fear of being infected and developing severe COVID-19 outcome or they might isolate themselves and make adverse changes in lifestyle behaviors (e.g., decreased physical activity, alcohol drinking) as a consequence of perceiving the risk and stress associated with having chronic physical conditions. Such psychological stress might further exacerbate their physical conditions and affect mental health. Thus, this finding underscores the urgency that we should be aware of their conditions and pay further attention for both physical and psychological care management and treatment for people with chronic physical conditions.

**Journal Requirements**

1. Please ensure that your manuscript meets PLOS ONE's style requirements, including those for file naming.

**Response:** We revised our manuscript following PLOS ONE’s style requirements.

2. Please include additional information regarding the survey or questionnaire used in the study and ensure that you have provided sufficient details that others could replicate the analyses. For instance, if you developed a questionnaire as part of this study and it is not under a copyright more restrictive than CC-BY, please include a copy, in both the original language and English, as Supporting Information.

**Response:** We provided information regarding the survey and questionnaire used in the present study in the method section while the questionnaire is copyrighted and thus is not publicly available.

3. Thank you for stating the following financial disclosure:“The study was supported by NCGM COVID-19 Gift Fund and the Japan Health Research Promotion Bureau Research Fund (2020-B-09).” Please state what role the funders took in the study. If the funders had no role, please state: ""The funders had no role in study design, data collection and analysis, decision to publish, or preparation of the manuscript."" If this statement is not correct you must amend it as needed. Please include this amended Role of Funder statement in your cover letter; we will change the online submission form on your behalf.

**Response:** We included the following statement in the financial disclosure section.

Line 248–251:

**Financial Disclosure**

The study was supported by NCGM COVID-19 Gift Fund and the Japan Health Research Promotion Bureau Research Fund (2020-B-09). The funders had no role in study design, data collection and analysis, decision to publish, or preparation of the manuscript.

4. In your Data Availability statement, you have not specified where the minimal data set underlying the results described in your manuscript can be found.

**Response:** We provide the following statement regarding data availability.

The data are not publicly available due to ethical restrictions for public deposition but available from the Department of Epidemiology and Prevention, Center for Clinical Sciences, National Center for Global Health and Medicine, Tokyo, Japan (Dr. Mizoue, mizoue@hosp.ncgm.go.jp).

5. We note that you have indicated that data from this study are available upon request. PLOS only allows data to be available upon request if there are legal or ethical restrictions on sharing data publicly.

**Response:** We provide the following statement regarding data availability.

The data are not publicly available due to ethical restrictions for public deposition but available from the Department of Epidemiology and Prevention, Center for Clinical Sciences, National Center for Global Health and Medicine, Tokyo, Japan (Dr. Mizoue, mizoue@hosp.ncgm.go.jp).

6. Please include a separate caption for each figure in your manuscript.

**Response:** We provided a caption for the figure 1 in our manuscript.

Line 165–166: The combined association of chronic physical condition(s) and SARS-CoV-2 infection risk at work with depressive symptoms is shown in Fig 1.

Line 177: Fig 1. The combined association of chronic physical condition(s) and degree of possible exposure to SARS-CoV-2 at work with depressive symptoms.
